# Generation of equine enteroids and enteroid-derived 2D monolayers that are responsive to microbial mimics

**DOI:** 10.1186/s13567-021-00976-0

**Published:** 2021-08-14

**Authors:** Stina Hellman

**Affiliations:** Department of Biomedical Sciences and Veterinary Public Health, Swedish University of Agricultural Sciences, SLU, P.O. Box 7028, 750 07 Uppsala, Sweden

**Keywords:** Equine, Enteroid, Enteroid-derived, Epithelium, 2D monolayer, Intestine, In vitro, TLR

## Abstract

Enteroid cultures are three-dimensional in vitro models that reflect the cellular composition and architecture of the small intestine. One limitation with the enteroid conformation is the enclosed lumen, making it difficult to expose the apical surface of the epithelium to experimental treatments. The present study was therefore conducted to generate cultures of equine enteroids and to develop methods for culture of enteroid-derived cells on a two-dimensional plane, enabling easy access to the apical surface of the epithelium. Equine enteroids were established from small intestinal crypts within 7–9 days of culture. Transcriptional analysis of cell type markers confirmed the presence of enterocytes, stem-, Paneth-, proliferative-, enteroendocrine-, goblet- and tuft cells. This cellular composition was maintained over multiple passages, showing that the enteroids can be kept for prolonged periods. The transfer from 3D enteroids to 2D monolayers slightly modified the relative expression levels of the cell type markers, indicating a decrease of goblet- and Paneth cells in the monolayers. Stimulation with the TLR2, 3 and 4 agonists Pam3CSK4, Poly I:C and LPS, respectively, induced the pro-inflammatory cytokines TNF-α and IL-8, while the TLR5 agonist FliC only induced TNF-α. In addition, an up-regulation of TGF-β, IL-33 and IFN-β was recorded after exposure to lipofected Poly I:C that also affected the monolayer integrity. Thus, the equine enteroid-derived 2D monolayers described in the present study show both genetic and functional similarities with the equine intestine making it an interesting in vitro model for studies demanding access to the apical surface, e.g. in studies of host-microbe interactions.

**Supplementary Information:**

The online version contains supplementary material available at 10.1186/s13567-021-00976-0.

## Introduction

Recent developments in stem cell technology have enabled three-dimensional in vitro culture of primary intestinal cells, commonly referred to as organoids. The organoid technology utilises the inherent ability of the intestinal epithelium to self-renew continuously and differentiate to specialised intestinal cell types. Propagation of organoids from the small intestine, referred to as enteroids, is enabled by crypt isolation and subsequent propagation and differentiation of stem cells in a three dimensional matrix supported by a cocktail of intestinal niche growth factors. Compared to immortalized cell lines, primary enteroid cultures display closer genetic and physiological similarities to the intestine in vivo [1, 2]. Thus, enteroids are a suitable in vitro model for studies of host-microbe interactions, inflammatory conditions and barrier effects of the intestine [2].

While enteroid cultures are starting to become standardized in human and mouse [3], there are less studies published on enteroids from farm animals as recently reviewed [4, 5] and only two studies describing equine enteroids [6, 7]. Several of the horse’s most common diseases are linked to the gastro-intestinal tract, such as colic, laminitis, diarrhoea and parasite infections. To my knowledge, there is no equine intestinal cell line commercially available and primary cultures from resections have a very limited survival time in vitro [8]. Arguably, development of the enteroid technology for equines will contribute as an essential tool for in vitro elucidation of the cause, as well as suggest preventions, to disorders affecting the intestinal tract of the horse.

One limitation with the organoid model is the difficulty to access the luminal surface of the epithelium which is enclosed within the organoid structure. To overcome this problem, methods for culturing organoid-derived cells on a two-dimensional plane have been elaborated for some species [9–13] but has not yet been described for equine enteroids. In monolayer culture systems, microbial antigens can easily be introduced to the apical surface of the epithelium making it a suitable model to study host-microbe interactions.

The intestinal epithelium can express a diversity of pattern recognition receptors (PRRs) that enables it to sense the presence of microorganisms and elicit pro- or anti-inflammatory responses [14, 15]. However, PRRs such as Toll-like receptor (TLR)-2, TLR4 and TLR5 seem to have an uneven spatial distribution along the intestinal tract that differs between species [16, 17]. In addition, RNA expression of TLRs does not always correlate with signal transduction capacity, addressing the importance of functional testing in the species of interest [17].

The aim of the present study was therefore to set up and characterize equine enteroids and enteroid-derived 2D monolayer cultures. The ability of the monolayers to produce pro- or anti-inflammatory reactions was assessed by exposure to TLR agonists followed by transcriptional analysis of cytokine genes.

## Materials and methods

### Horses

Intestinal tissue was collected from three Swedish warmblood mares, aged 10–14 years, owned by the Department of Clinical Sciences, SLU, Uppsala, Sweden. The horses were euthanized for reasons unrelated to the study and two of the horses had been anesthetized for 4–5 h prior to sampling. Intestinal tissue from all horses was collected within one hour after euthanasia (Ethical permit 5.8.18–15533/2018).

### Crypt isolation

Isolation of crypts from the small intestine and the subsequent culture of equine enteroids were performed in accordance with a previously published protocol [6] with slight modifications. To isolate crypts, 15–20 cm sections of equine mid-jejunum were collected. The tissue sections were opened longitudinally and washed in ice-cold phosphate buffered saline (PBS; SVA, Uppsala, Sweden) containing antimicrobials, i.e. 200 IU/mL penicillin, 100 µg/mL streptomycin (Invitrogen, CA, USA) and 1 µg/mL Amphotericin B (Thermo Fisher Scientific, UK) before sectioned into 1–2 cm pieces. The sections were incubated in 50 mL tubes containing PBS with 30 mM EDTA (Invitrogen, CA, USA), 1 mM DTT and 10 µM Y-27632 (BD Biosciences, USA) on ice and placed on an orbital shaker platform moving at 60 rpm for 30 min with increased shaking every fifth minute. Thereafter, the tissue sections were moved to a fresh 50 mL tube containing PBS with 30 mM EDTA and 10 µM Y-27632 pre-warmed to 37 °C and incubated for 10 min on a shaker at 60 rpm, 37 °C. To free crypts from the epithelium, the tissue pieces were incubated for 5 min in ice-cold PBS containing antimicrobials, while vigorously shaken. After each washing the recovery of villi and crypts was examined by light microscopy. This step was repeated until crypts dominated over villi in the solution with a minimal contamination of debris. The wash solution containing most crypts was filtered through a 100-micron cell strainer (Sarstedt) to remove remaining villi.

### Enteroid culture

The crypts were pelleted by centrifugation for 5 min at 200×*g* and suspended in ice-cold Matrigel™ GFR Membrane Matrix (Corning™) containing recombinant human growth factors; 100 ng/mL Noggin (R&D systems), 500 ng/mL R-spondin (Peprotech), 50 ng/mL EGF (Corning™), 100 ng/mL Wnt3a (R&D Systems™), 10 µM Y-27632, 10 µM SB202190 (Tocris Bioscience™), 500 nM LY2157299 (A ChemBlock) and 2.5 µM CHIR99021 (Tocris Bioscience™), at a concentration of 20–40 crypts per 50 µL Matrigel. Thereafter, 50 µL aliquots were plated on a pre-warmed 24-well plate (Nunclon delta, Thermo Scientific™) and set to polymerize at 37 °C. After 30 min incubation, 0.5 mL over-lay medium, i.e. DMEM/F12 containing 1 × GlutaMAX (Gibco™) and 1 × N-2 supplement (Gibco™), 1 × B-27 supplement (Gibco™), 200 IU/mL penicillin, 100 µg/mL streptomycin and 10 mM HEPES (Invitrogen, CA, USA), was carefully added on top of the Matrigel domes and incubated at 37 °C in 7% CO_2_. Growth factors were added every second day and the entire volume of over-lay medium was changed every fourth day.

### Passage of enteroids

**Figure 1:**
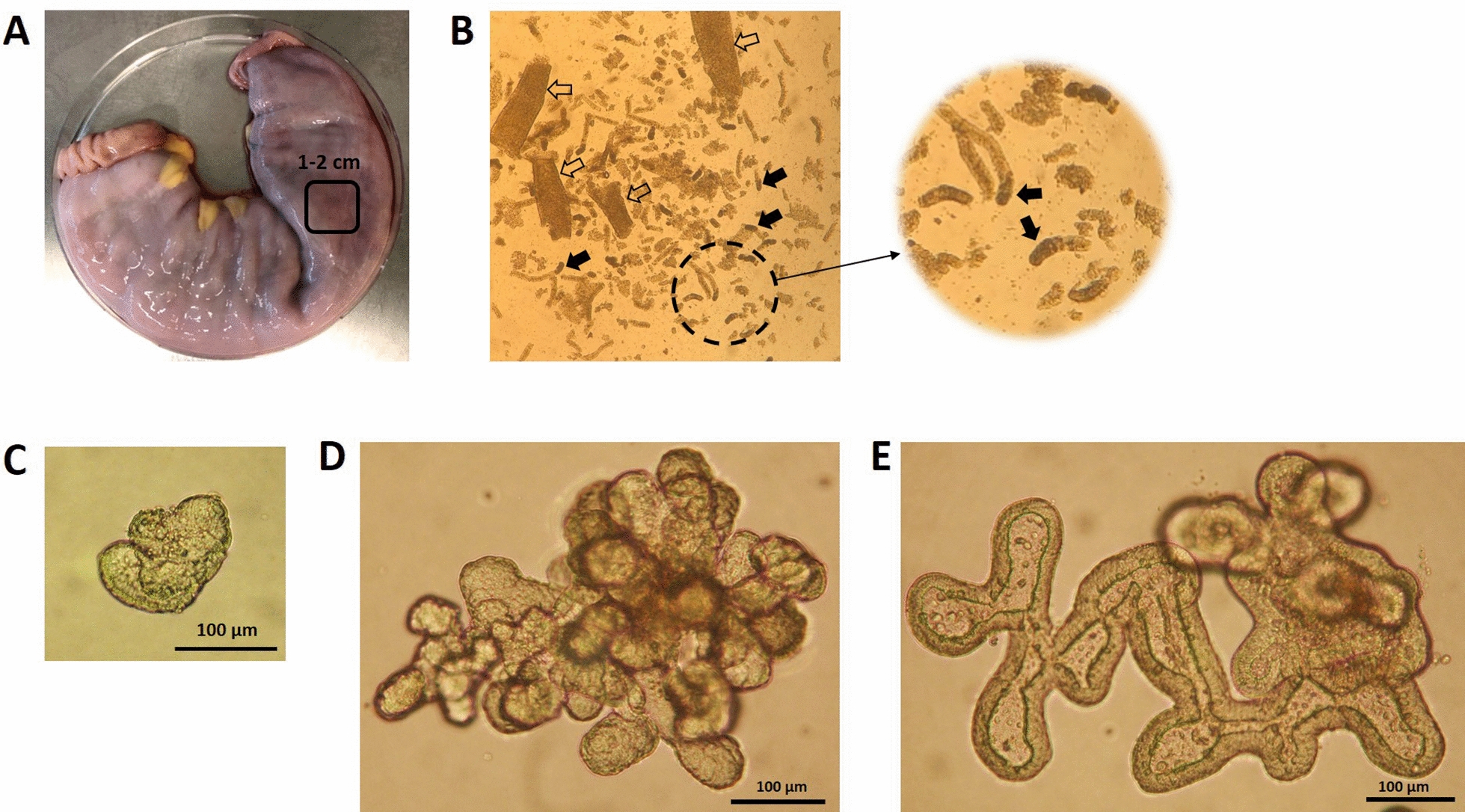
**Scheme for isolation of crypts from equine jejunum and subsequent 3D culture.A** Section of equine jejunum. **B** Recovered villi (open arrows) and crypts (closed arrows). **C** Spheroid after four days of culture. **D** Enteroid after seven days of culture. **E** Enteroid at four days of culture after the third passage. Representative results from one of three horses. For details see Material and Methods (Photo: Nikon Coolpix 990).

To avoid loss of material as both crypts and enteroids easily attached to surfaces, all pipette tips and tubes were pre-wetted in ice-cold phosphate buffered saline (PBS) containing 0.1% bovine serum albumin (PBS + BSA) prior to usage. Once the enteroids demonstrated significant budding and growth (Figure 1) they were passaged by fragmentation into new crypt units. Briefly, the over-lay medium was removed and the domes were carefully washed three times in 37 °C PBS. The enteroids were detached by addition of one mL ice-cold Cell Dissociation Solution (Corning™) to each well followed by mechanical disruption of the Matrigel dome using a 100–1000 µL pipette. The solution containing enteroids was transferred to a 10 mL tube and incubated on ice for 10 min. The enteroids were thereafter washed three times in PBS + BSA by centrifugation for 5 min at 200 × *g*. Before the final wash the enteroid pellet was suspended in 2 mL PBS + BSA and fragmented by pipetting 15–20 times. The level of fragmentation was examined in 50 µL aliquots by light microscopy. If required, additional pipetting was performed until most enteroids were fragmented. The fragments were thereafter suspended in growth factor supplemented Matrigel and plated as described above.

### Freeze preservation of crypts and enteroids

Excess crypts, enteroids or fragmented enteroids were frozen in DMEM/F12 containing 10% dimethyl sulfoxide (DMSO; Sigma-Aldrich) in cryo tube vials (Nunc™, Denmark) placed in a MrFrosty™ freezing container (Thermo Scientific™) ensuring a slow rate of cooling in a −80 °C freezer. To thaw, the samples were warmed at 37 °C. The thawed cell suspensions were transferred to 10 mL tubes and washed three times in PBS-BSA prior to plating.

### Enteroid-derived 2D monolayer culture

Enteroids at day 4 of culture were used to establish 2D monolayers according to a previously published protocol for porcine cells [12]. The enteroids were detached from the Matrigel domes by incubation in 1 mL ice-cold DMEM/F12 for 10 min on ice. The enteroids were transferred to a 50 mL tube and pelleted for 5 min at 200 × *g* and thereafter suspended in 5 mL 1 × TrypLE Express Enzyme (Gibco™). After 10 min incubation at 37 °C the enteroids were disrupted to single cells by pipetting. Trypsination was stopped by adding four volumes of DMEM/F12 containing 5% fetal calf serum (FCS; Invitrogen, CA, USA), 10 µM Y-27632 and antimicrobials, followed by centrifugation for 5 min at 450 × *g*. The cells were suspended in monolayer growth medium, i.e. DMEM/F12 containing 5% FCS, 10 mM HEPES, 1 × N-2, 1 × B-27, antimicrobials and organoid growth factors. Between, 40–50 000 cells/cm^2^ were seeded in pre-coated 24-well plates or in polycarbonate transwell cell culture inserts for 12-well plates with a pore size of 0.4 micron (Thermo Scientific™ Nunc™). Plates and inserts were pre-coated with 200 µL of Matrigel and DMEM/F12 mixed at a ratio of 1:30. The plates were incubated for 1–2 h at 37 °C before the excess medium was aspirated and the plates were let to air-dry under a fume hood for 10 min prior to use. Volumes of monolayer growth medium used for the 24-well plates and 12-well transwell inserts were 0.5 mL and 2 mL (0.5 mL on top + 1.5 mL below), respectively. The enteroid monolayer cultures were incubated up to 15 days at 37 °C with 7% CO_2_ in the air, exchanging the medium every second day. The epithelial integrity in the transwell cultures was monitored daily by measuring the trans-epithelial electrical resistance (TEER) using an Epithelial Volt-Ohm Meter (Millicell ERS-2, Millipore). The resistance per cm^2^ (Ω.cm^-^^2^) was calculated based on the surface area of the inserts (1.13 cm^2^).

### Enteroid-derived 2D monolayer exposure to TLR agonists

Various concentrations of LPS from *E. coli* O111:B4 (1, 5 or 10 µg/mL; Sigma), Pam3CSK4 (0.5, 1.5 or 4.5 µg/mL; InvivoGen), FliC (0.1, 0.3 or 0.9 µg/mL; VacciGrade™, InvivoGen) or Poly I:C (10, 20 or 30 µg/mL; P-L Biochemicals, Inc) were tested to determine the optimal cytokine-inducing concentration for each TLR agonist in the enteroid-derived monolayers. The experiments were conducted using two-day old enteroid-derived 2D monolayers, cultured in 24-well flat-bottomed plates, with a total stimulation time of 20 h. Poly I:C was pre-incubated with Lipofectin (2.5 µg/mL; Invitrogen, CA, USA) 15 min before addition to the monolayers. To control for cytopathic effects, lipofectin was added at various concentrations (0.1, 0.25, 0.5, 1.5 or 2.5 µg/mL) to the 2D monolayers in the absence of Poly I:C. The cell integrity was monitored after 20 h of incubation and gene expression analysis was performed on cells incubated with 2.5 µg/mL lipofectin. Each stimulation was performed in quadruplicates and parallel enteroid-derived 2D monolayers cultured in plain growth medium were used as controls. After 20 h incubation, the monolayers were harvested in Trizol reagent (Invitrogen, CA, USA) and stored at -80 °C until use.

### RNA isolation and cDNA synthesis

qPCR analysis was performed on 3D enteroids and 2D monolayers collected at day 4 and 3 of culture, respectively. RNA was extracted using a combined Trizol and column-based protocol (E.Z.N.A total RNA kit, Omega Biotek) as previously described [18]. The quantity and purity of the RNA was measured by spectrophotometry (NanoDrop ND-1000, NanoDrop Technologies). To eliminate genomic DNA contamination each RNA was treated with RQ1 RNAse-free DNAse (Promega). Synthesis of cDNA was performed using 1.2 μg of RNA (GoScript Reverse transcription system; Promega) and a –RT control was run in parallel. The samples were diluted 1:5 and stored at -20 °C until use.

### Characterisation of enteroids and enteroid-derived 2D monolayers

Primer pairs for detection of genes representative for epithelial cells (EPCAM), proliferative cells (PCNA and SOX9), stem cells (LGR5 and SOX9), Paneth cells (LYZ) enteroendocrine cells (CGA), goblet cells (MUC2), and tuft cells (DCKL1) were used to characterise enteroids and the monolayers by qPCR (Additional file 1). Each primer pair was optimised for the assay specific concentration and annealing temperature using reference cDNA made from sections of equine intestine [19]. TLR activation was estimated using primer pairs for equine IFN-α, IFN-γ, IL-1β, IL-4, IL-5, IL-6, IL-8, IL-9, IL-10, IL-13, IL-17A, TGF-β, TLR4 and TNF-α using previously established conditions [18, 20, 21] and for equine IFN-β, IL-33, TLR2, TLR5 and TSLP using conditions summarized in Additional file 1.

Duplicates of 2 µL cDNA in 23 µL Quantitect SYBR Green PCR mix (Qiagen) were run in a CFX96 Touch PCR machine (Bio‐Rad). The run protocol was an initial 15 min cycle of 95 °C followed by 40 cycles of 95 °C for 15 s, the assay specific annealing temperature for 30 s and 72 °C for 30 s ending with a melt curve analysis to verify the PCR product. To enable relative quantifications, primer pairs for seven reference genes; β2M, GAPDH, H2A type 1, HPRT, RPL32, SDHA and TFRC that have previously been evaluated in multiple sections of the equine intestine [19], were tested for their expression stability in two batches of enteroids and two batches of transwell monolayers using the geNorm software (qBase^PLUS^, Biogazelle). Consistent with previous data on sections of equine jejunum [19] the genes GAPDH (M = 0.856; CV = 0.239), HPRT (M = 0.802; CV = 0.237) and SDHA (M = 0.899; CV = 0.301) were most stably expressed and were therefore selected for normalisation of data. The results are expressed as 2^−ΔCt^ or fold change as indicated in the graphs. Fold change value for the gene of interest was calculated in relation to the calibrator gene after normalisation to the geometric mean for the reference genes. Genes with fold change > 2 were considered as up-regulated.

### Preparation of cross-sections and HE-staining

The enteroids were prepared for hematoxylin and eosin (HE) staining as previously described for human colonoids [22]. Briefly, the enteroids were fixated for 30 min in 4% paraformaldehyde. To increase the number of enteroids on each cross-section, multiple enteroid domes were dissociated by gentle pipetting and pooled into a 50 mL tube. The enteroids were pelleted by centrifugation for 5 min at 250 × *g* and suspended in pre-warmed 2% agarose (Type IX, Sigma-Aldrich). To accumulate the enteroids onto a single plane, the tube was immediately centrifuged for 3 min at 150 × *g*. The agarose was solidified on ice, embedded into paraffin wax and sectioned. This treatment allowed staining for histological examination (Additional file 2).

### Statistical analysis

Statistical analysis was made using Prism 7.0 (GraphPad software). Differences in the expression of cell lineage- and cytokine genes between enteroids and enteroid-derived 2D monolayers were calculated on ΔCt and ΔΔCt values, respectively, using the one-way ANOVA followed by Tukey’s multiple comparison test. *P*-values below 0.05 were regarded as significant. When indicated, variability of gene expression data is reported as mean ± SD.

## Results

### Establishment of enteroid-derived 3D cultures from equine intestine

Crypts were isolated from mid-jejunal sections sampled from horses within one hour after euthanasia (Figures 1A, B). Two of the three horses had been anesthetised during surgery for 4–5 h and crypts from these horses were released earlier (wash 3–4) compared to the third horse (wash 7–9). In addition, the enteroid plating efficiency was less (~20–25%) for the anesthetised horses compared to the horse that had not been subjected to anaesthesia (~80%). Regardless, crypts from all horses started to form a lumen and bud structures after 3–4 days in culture (Figure 1C). After 7–10 days, the enteroids had developed more complex structures with a lumen enclosed by a distinct epithelium and crypt-like buds (Figures 1D, E). At day 9–12 of culture the core of the enteroids gradually darkened, indicating accumulation of dead cells in the lumen. The optimal time to split the enteroids was therefore set to 7–9 days after crypt isolation.

**Figure 2:**
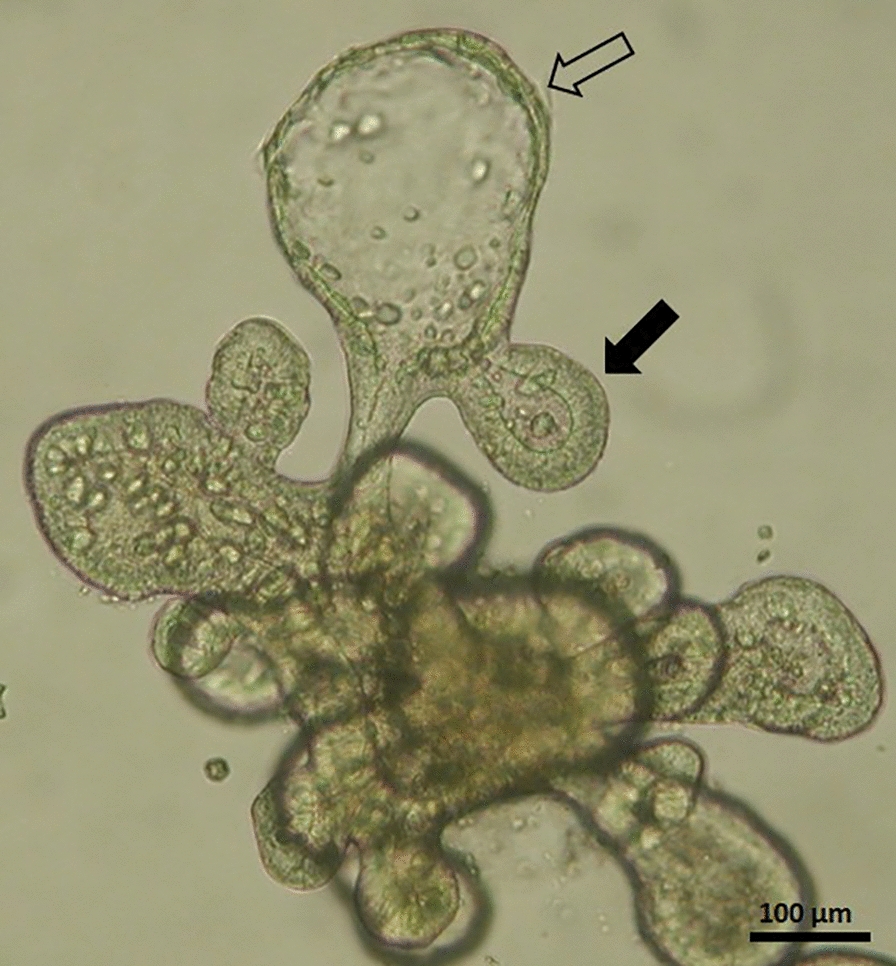
**Altered morphology of an equine enteroid cultured at suboptimal conditions.** Enteroid displaying both normal morphology (closed arrow) as well as circular buds with a thinned epithelium (open arrow), suggesting an increased rate of apoptosis without compensatory proliferation (Photo: Nikon Coolpix 990).

The enteroids could successfully be passaged by fragmentation into new crypt-like structures at an optimal split rate of 1:8. After splitting, enteroids were re-generated within 4–5 days of culture and were split every 4^th^ day thereafter. The optimal seeding density was found to be 20–40 fragments per Matrigel dome. At denser seeding, the enteroids gained a circular morphology with a thinned epithelial layer (Figure 2). This morphology indicates a condition of increased cell death without compensatory cell proliferation, as previously described in murine enteroids [23]. Both intact and fragmented enteroids could be frozen at −80 °C and brought up in culture with 80–90% recovery. The corresponding figure for frozen crypts ranged from 20–50% recovery.

### Analysis of epithelial cell lineages in 3D equine enteroids

**Figure 3:**
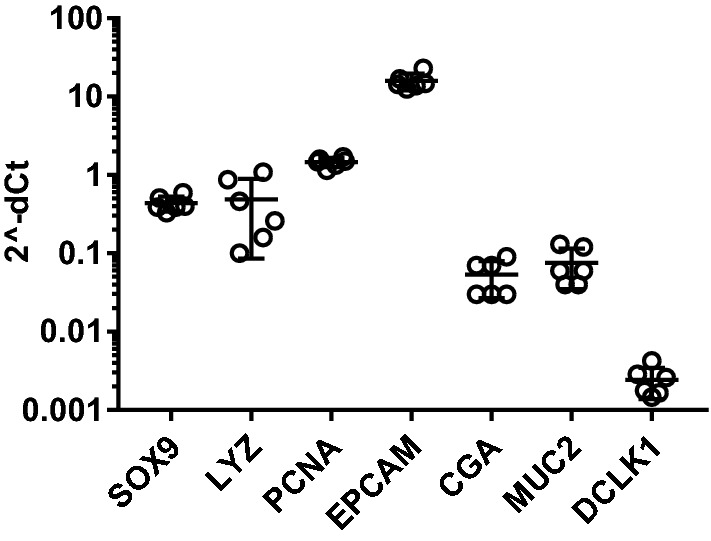
**Stability of equine enteroid cellular composition over six passages.** Expression of the cell lineage markers SOX9, LYZ, PCNA, EPCAM, CGA, MUC2 and DCLK1 in six succeeding passages of enteroids established from one horse. Each passage is represented by an open circle. The gene expression was normalized to the geometric mean for the reference genes (HPRT, SDHA and GAPDH) and presented as 2^−ΔCt^.

Genetic markers present in intestinal stem cells (SOX9), Paneth cells (LYZ and SOX9), proliferative cells (SOX9 and PCNA), epithelial cells (EPCAM), enteroendocrine cells (CGA), goblet cells (MUC2) and tuft cells (DCLK1) were all verified in enteroids established from the three horses (Figure 3). The expression levels of these markers were sustained through six passages demonstrating that the enteroid cultures can be passaged repeatedly and maintained for a longer period without compromising the cellular composition. The highest expression was consistently recorded for EPCAM confirming that epithelial cells dominated the enteroids (Figure 3). The presence of LGR5 expressing cells was only indicated and the basal expression level of LGR5 seemed to be very low, yielding Cq values > 35 with occasionally undetected technical replicates.

### Transfer of equine enteroids to 2D monolayer cultures

**Figure 4:**
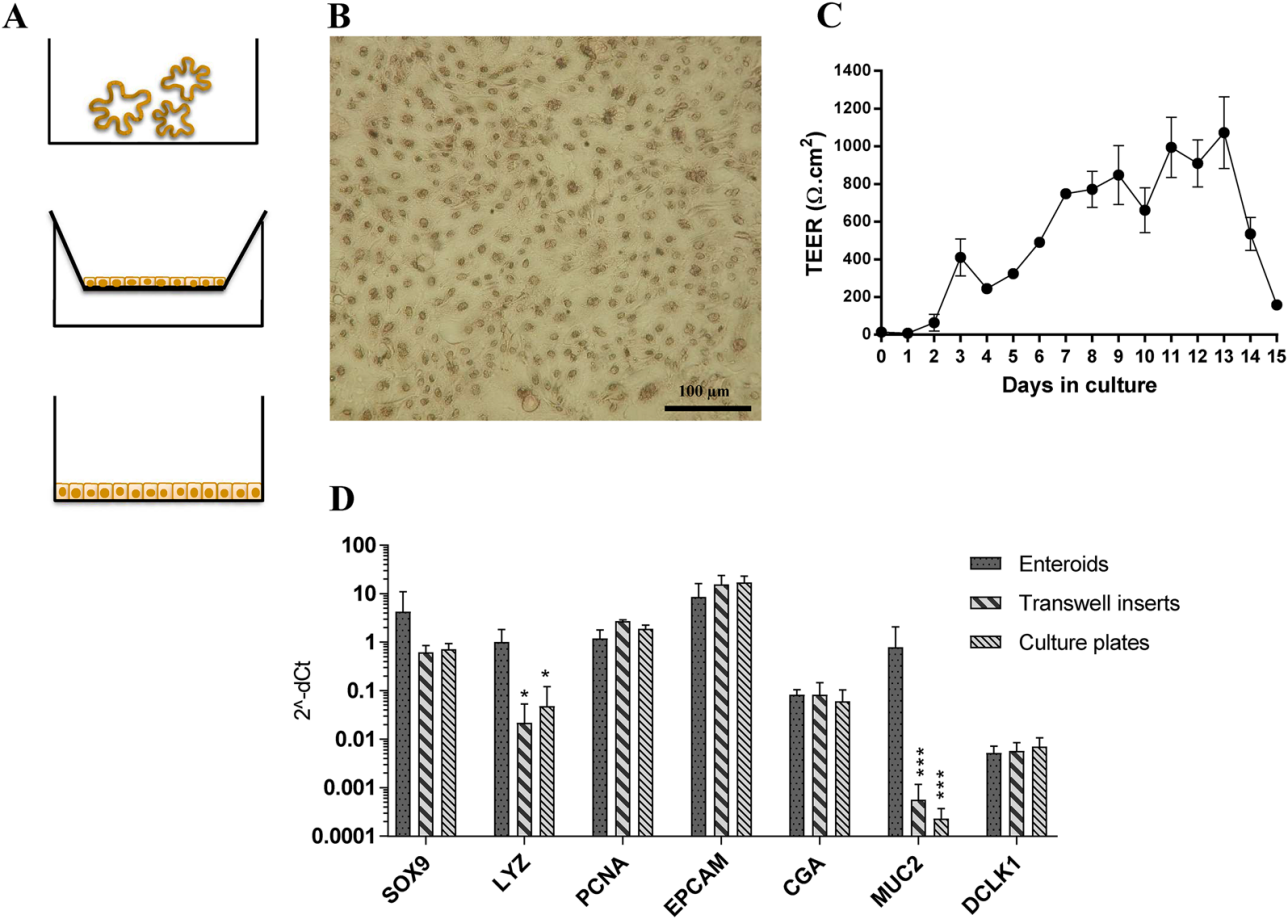
**Cellular composition after transfer of equine enteroids to 2D monolayers**. Enteroids could be dissociated to single cells and re-established on pre-coated plates or transwell inserts. **A** Graphical illustration of enteroid and monolayer cultures. **B** Monolayer after three days of culture on a flat-bottomed plate. Contrast stained by hematoxylin (Photo: Nikon D40x). **C** Transepithelial electrical resistance in transwell cultures monitored over 15 days (mean value ± SD, two technical replicates). **D** Expression of the cell lineage markers SOX9, LYZ, PCNA, EPCAM, CGA, MUC2 and DCLK1 in enteroids or in 2D monolayers cultured on plates or transwell inserts. The gene expression was normalized to the geometric mean for the reference genes (HPRT, SDHA and GAPDH) and is presented as 2^−ΔCt^. The results are given as mean values ± SD for cultures established from each of the three experimental horses (*n* = 3). *P* < 0.05 = * and *P* < 0.001 = ***.

To obtain two-dimensional cultures, the enteroids were dissociated to single cells and seeded on transwell inserts or on flat-bottomed culture plates pre-coated with Matrigel (Figure 4A). After three days of culture, more than 90% confluence was reached (Figure 4B). Trans-epithelial electrical resistance analysis of the transwell cultures showed that the monolayer integrity was stabilised after one week and remained so for another week with an average TEER value of 860 ± 160 Ω.cm^-^^2^. After 13 days of culture the TEER rapidly decreased (Figure 4C). In comparision, the 2D monolayers cultured on flat-bottomed plates remained confluent for up to 8 days. Genomic characterisation verified that all cell types present in the enteroids were also established in the 2D monolayers, regardless of whether they were grown on transwell inserts or flat-bottomed culture plates. However, the expression of the markers for Paneth (LYZ) and goblet (MUC2) cells was significantly reduced in the 2D monolayer cultures compared with the enteroid cultures (Figure 4D).

**Figure 5:**
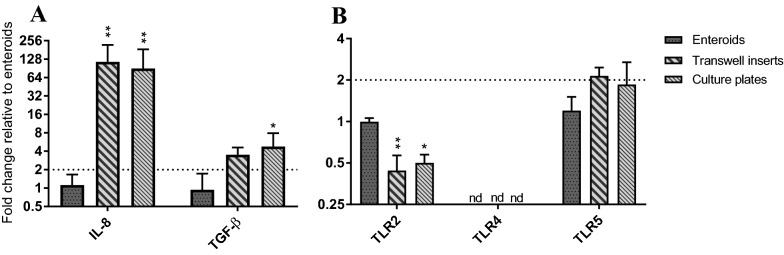
**Relative expression of IL-8, TGF-β and TLRs in equine enteroids and 2D monolayers**. The gene expression of IL-8, TGF-β (**A**), TLR2, TLR4 and TLR5 (**B**) in the monolayer cultures was normalised to the geometric mean for the reference genes (HPRT, SDHA and GAPDH) and calibrated to that in the enteroids. The results are given as mean values ± SD for cultures established from each of the three experimental horses (*n* = 3). *P* < 0.05 = * and *P* < 0.01 = **. FC > 2 shown by dashed line indicates gene up-regulation. nd = not detected.

The expression of cytokine genes and TLR receptors was also affected by the shift from 3D culture to 2D monolayers. A baseline expression of IL-5, IL-8, IL-33, TNF-α and TGF-β was detected in both the 3D enteroids and the 2D monolayers regardless if cultured on transwell inserts or culture plates. The genes encoding IFN-α, IFN-γ, IL-1β, IL-4, IL-6, IL-9, IL-10, IL-13, IL-17A and TSLP occasionally showed a low expression but in that case only in one of the two technical replicates. After transfer from enteroid morphology to 2D monolayers, the constitutive expression of IL-8 and TGF-β significantly increased whereas the expression of TLR2 was reduced. The expression of TLR5 remained unaltered and TLR4 was not detected in any of the culture systems (Figure 5).

### Cytokine response to TLR agonists in equine enteroid-derived 2D monolayers

To assess the responsiveness of equine enteroid-derived monolayer cultures to microbial stimuli, two-day-old monolayers were exposed to the TLR agonists Pam3CSK4 (TLR2/1), Poly I:C (TLR3), LPS (TLR4) or FliC (TLR5) for 20 h followed by transcriptional analysis of cytokine genes. The titrations showed a dose–response curve and the concentrations inducing the highest gene expression was chosen for each TLR-agonist; Pam3CSK4, 4.5 µg/mL; Poly I:C, 20 µg/mL; LPS, 5 µg/mL and FliC 0.1 µg/mL.

**Figure 6:**
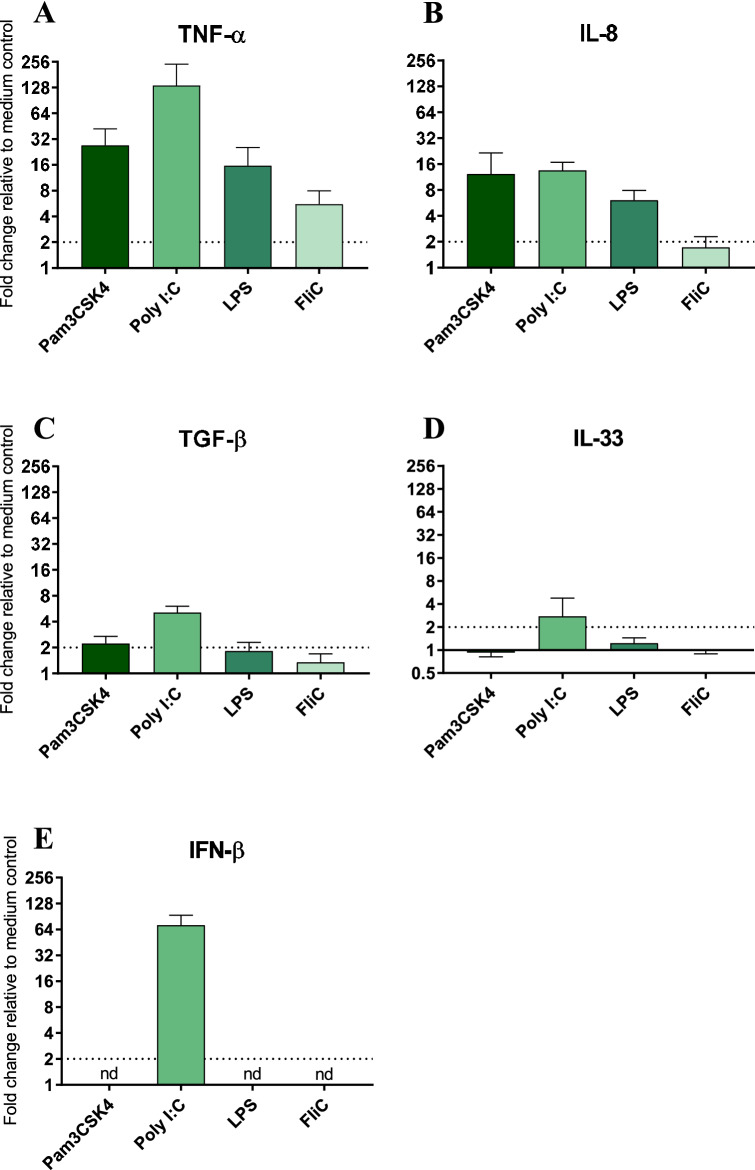
**Effects of TLR agonist on cytokine gene expression in equine enteroid-derived 2D monolayer cultures.** The relative gene expression of TNF-α (**A**), IL-8 (**B**), TGF-β (**C**), IL-33 (**D**) and IFN-β (**E**) in monolayer cultures exposed to Pam3CSK4, lipofected Poly I:C, LPS or FliC for 20 h. The cytokine gene expression was normalised to the geometric mean for the reference genes (HPRT, SDHA and GAPDH) and calibrated to that in the medium control. The results are given as mean values ± SD for cultures established from two of the experimental horses (*n* = 2). FC > 2 shown by dashed line indicates gene up-regulation. nd = not detected.

No morphological changes were observed in the presence of Pam3CSK4, FliC, LPS or at any of the lipofectin concentrations tested. In contrast, exposure to lipofected Poly I:C resulted in partly disrupted monolayers along with an increased amount of floating cells that were removed before processing for gene expression analysis (Additional file 3A, B). No induction of cytokine genes was recorded after exposure to lipofectin alone (Additional file 3C), but lipofected Poly I:C induced gene expression of TNF-α, IL-8, TGF-β, IL-33 and IFN-β (Additional file 3C and Figure 6). Pam3CSK4 and LPS induced a similar gene expression profile, shown by up-regulation of the genes encoding TNF-α and IL-8, but not TGF-β, IL-33 or IFN-β (Figure 6). The presence of FliC resulted in up-regulation of TNF-α but none of the other cytokines tested (Figure 6). The gene encoding IL-5 was not induced by any of the TLR agonists.

## Discussion

Experience from the set up of equine enteroid cultures and the subsequent development of a 2D monolayer model from these is described. Such a 2D system enables access to the apical surface of the equine intestinal epithelium making it an appropriate experimental system to study reactions at the intestinal host-microbe interface. In the present study, the equine enteroid-derived 2D monolayers were validated with regard to cell lineage gene expression and tested for the cytokine response to TLR agonists.

The enteroid plating efficiency differed between the three horses sampled with a reduced yield from the two horses that had been subjected to anaesthesia prior to sampling (~20 and 25% vs 80%). A similar range of inter-individual variation has previously been shown for equine enteroids established from five horses [6]. However, it cannot be excluded that an extended time of general anaesthesia has an effect on the intestinal function that impaired the recovery of enteroids in the present study. Regardless, it is important to note that differentiated enteroids with similar cellular composition developed within 7–9 days culture of crypts isolated from all three horses.

The cellular composition was determined by genomic characterisation of the enteroids and the enteroid-derived 2D monolayers. The presence of the cell lineages expected to be present in enteroids, such as stem cells, Paneth cells, enterocytes, enteroendocrine cells and goblet cells [1] was indicated. These results are consistent with previous characterisation of equine enteroids [6] demonstrating a good reproducibility of the methods used for isolation of equine crypts and subsequent enteroid culture. All cell lineage markers analysed were stably expressed for six succeeding passages showing that equine enteroid cultures can be maintained for multiple passages without compromising the cellular composition, as previously shown for porcine and bovine enteroids [24, 25]. In addition, a fifth epithelial cell lineage, tuft cells, was detected using primer pairs for the equine DCLK-1 gene, which is uniquely expressed by tuft cells in the intestinal epithelium. Tuft cells are multifunctional cells with chemosensory capabilities that are involved in tissue regeneration after injury, chronic inflammation, cancer initiation and immune responses against parasites [26]. Thus, these results indicate that the equine enteroids share the genetic signatures of the intestinal epithelium in vivo. In addition, the presence of tuft cells make the enteroids interesting models for studies of host-parasite interactions, which is a research area that is currently lacking good in vitro models [27].

The generation of enteroids is based on the proliferation and differentiation of stem cells from small intestinal crypts. In murine and human enteroids, intestinal stem cells are commonly characterized by their expression of LGR5, a Wnt target gene, which is selectively expressed at the base of intestinal crypts [28]. The basal expression level of LGR5 in the equine enteroids seemed to be very low. In accordance, analysis of archived samples from equine jejunum, ileum, caecum and colon [19] also indicated a low or undetectable basal expression of LGR5 (data not shown). This contradicts previous successful results using equine enteroids [6]. However, the LGR5 + stem cells constitute a very small fraction of murine intestinal epithelial cells [28]. Because most descriptions of intestinal stem cells are from studies of murine or human tissues [29], potential species-specific differences cannot be excluded. In addition to LGR5, the SOX9 gene is an important regulator of the Wnt signalling pathway and a key driver of epithelial turnover [30]. SOX9 is mainly expressed by stem and progenitor cells but also by Paneth cells [31]. Nevertheless, the high expression of SOX9 along with the efficient propagation of enteroids verified the presence and maintenance of stem cells in the equine enteroids.

One limitation with the enteroid model is the enclosed morphology, which makes it difficult to expose the apical surface to experimental treatments. To facilitate access to the lumen, enteroid-derived cells can be cultured as 2D monolayers. This approach has been especially useful in studies of host-microbe interactions [11, 32–34]. To enable these types of studies in equine enteroids, a simple protocol for culture of equine enteroid-derived 2D monolayers was adapted from porcine enteroids [12]. By enzymatically dissociating the enteroids to single cells in the presence of a Rho kinase (ROCK) inhibitor Y-27632 the enteroid-derived cells could be seeded on Matrigel pre-coated culture plates or on transwell inserts with minimal cell mortality. With a seeding concentration of 40 000–50 000 cells/cm^2^, confluent monolayers were obtained after three days of culture, making it an effective model suitable for high-throughput screenings.

Genomic characterization of the monolayers confirmed the presence of all cell lineages found in enteroids, regardless if cultured on a flat-bottomed plate or on a transwell insert. However, the expression LYZ and MUC2 was significantly reduced in the monolayer cultures compared to the enteroids, indicating a loss of Paneth- and goblet cells, but with retained SOX9 expression. Cell proliferation and differentiation along the crypt-villus axis is coordinated by a number of signaling pathways influenced by Wnt factors that promotes stem cell proliferation [35]. When the intestinal epithelium loses its 3D conformation, as when cultured in 2D monolayers, this Wnt regulation might also be lost. In accordance, several methods have been elaborated for differentiation of enteroid monolayers concluding that a reduction of Wnt stimulating factors, such as Wnt3a and R-spondin, promotes differentiation of goblet cells [9, 10, 33]. The growth medium used in the present study for equine 2D monolayers was supplemented with Wnt3a and R-spondin, likely explaining the observed decrease of goblet cells. Moreover, a recent study performed on murine enteroid monolayers described a feed-back loop where elevated Wnt factors down-regulated the number of Paneth cells [36], possibly explaining the reduced expression of LYZ in the equine 2D monolayers. Accordingly, there is a potential to optimize the growth factor conditions needed to generate equine enteroid-derived 2D monolayers supporting differentiation of Paneth and goblet cells.

The transfer from 3D enteroids to 2D monolayers was accompanied by a strong increase in the IL-8 gene expression. In the intestine, IL-8 is one of the first cytokines released by injured or inflamed epithelium, and is constitutively expressed by multiple intestinal epithelial cell lines [37]. Accordingly, high basal protein levels of IL-8 have been observed in transwell cultures of human enteroids [11]. Despite that, the expression of IL-8 could be further enhanced by exposure to external stimuli.

The defined compounds, Pam3CSK4, Poly I:C, LPS and FliC were used to mimic microbial insults. As a read out, a broad panel of qPCR assays detecting immune-related genes that represent Th1, Th2, Th17, Th9 and Treg responses was used. Out of the tested TLR agonists, the RNA analogue Poly I:C was the most potent inducer of the pro-inflammatory cytokine genes TNF-α and IL-8, and the only compound to up-regulate TGF-β, IL-33 and IFN-β. Notably, exposure to Poly I:C impaired the monolayer integrity, potentially explaining the induction of TGF-β and IL-33 which are two immune-regulatory cytokines released after cell injury [38, 39]. These observations emphasize the relevance of equine enteroid-derived 2D cultures in future studies of invasive pathogens that are known to break the epithelial barrier. The bacterial compounds Pam3CSK4, LPS and FliC all gave a moderate up-regulation of TNF-α while Pam3CSK4 and LPS also up-regulated IL-8. Together, these results show that the equine enteroid-derived 2D monolayers can respond with similar pro- and anti-inflammatory reactions upon exposure to microbial structures as would be expected in vivo [15].

Recognition of microbial structures is mediated by PRRs at the apical surface of intestinal epithelial cells. As intestinal epithelial cells are the first to encounter microbial antigens, this recognition and the subsequent induction of cytokines and chemokines are essential for recruitment and activation of immune cells. However, given the constant exposure to the intestinal microbiota, the expression and signal transduction capacity of PRRs is tightly regulated to maintain homeostasis [40]. To estimate the expression pattern of selected TLRs in the present culture systems, enteroids and 2D monolayers were analysed for the presence of TLR 2, 4 and 5. In both culture systems, the genes encoding TLR2 and TLR5, but not TLR4 were detected. However, the expression of TLR2 was significantly lower in the 2D monolayers compared to enteroids, potentially reflecting the reduced presence of Paneth cells in the monolayers. The undetectable gene expression of TLR4 is in line with previous observations in murine and human enteroids [16, 17]. Still, exposure to LPS induced TNF-α and IL-8, suggesting activation via the cytosolic receptor caspase-11 or via the transient receptor potential (TRP) ion channels on the cell surface [41, 42].

In conclusion, the equine enteroid cultures presented in this study display a cellular composition and architecture that largely reflects the intestine in vivo, making it a relevant in vitro model for studies dealing with the equine intestine. For applications demanding contact with the apical surface of the epithelium, enteroid-derived cells can be cultured as 2D monolayers that show similar genetic features as the enteroids. The equine enteroid-derived 2D monolayers are responsive to stimulation with microbial mimics, opening up for their use in future studies of host-microbial interactions in the equine intestine.

## Supplementary Information

**Additional file 1. Table of primer pairs.** Primer details and optimized qPCR conditions.

**Additional file 2. HE image of equine enteroids.** HE stained section of equine enteroids at day 7 of culture. Arrows indicate goblet cells.

**Additional file 3. Equine enteroid-derived 2D monolayers exposed to Poly I:C + Lipofectin (A) or Lipofectin alone (B) and the relative expression of cytokine genes (C) after 20 h stimulation.** The figure shows data from one representative horse (Photo: Nikon Coolpix 990).

## Data Availability

The data that support the findings of this study are available on request from the corresponding author.

## Abbreviations

TLR: Toll-like receptor
PRR: Pattern recognition receptor
BSA: Bovine serum albumin
LPS: Lipopolysaccharide
HE: Hematoxylin and eosin
HMG: High mobility group
TEER: Trans-epithelial electrical resistance
