# Supplementary material for: Generation of equine enteroids and enteroid-derived 2D monolayers that are responsive to microbial mimics
Source: Vet Res. 2021 Aug 14;52:108. doi: 10.1186/s13567-021-00976-0 (PMC8364015; doi:10.1186/s13567-021-00976-0)
Supplement: Supplementary file 1 — Additional file 1. Table of primer pairs. Primer details and optimized qPCR conditions. [file 13567_2021_976_MOESM1_ESM.docx]

Additional file 1. Table of primer pairs

| **Target** | **Gene** | **Primer sequence** | **Primer conc. (nM)** | **Annealing temp. (⁰ C)** | **Melting temp. (⁰ C)** | **Efficiency (%)** | **r^2^** | **Source** |
| --- | --- | --- | --- | --- | --- | --- | --- | --- |
| Epithelial cells | EPCAM | F: TGA CCA CAA ACT GCT CTG TGA | 400 | 59 | 80 | 94 | 0.999 | [6] |
|  |  | R: GAG CCC GTC GTT ATT CTG GAT |  |  |  |  |  |  |
| Proliferative cells | PCNA | F: AAG CCA CTC CAC TGT CTC CTA | 400 | 59 | 77.5 | 96.9 | 0.998 | In house |
|  |  | R: CTA GGA TGC GTC CTC ATC CTC |  |  |  |  |  |  |
| Intestinal stem cells | LGR5 | F: AGC CTG GTG GTT CTG CAT CT | 400 | 57 | 78 | * |  | [6] |
|  |  | R: AAC GCT TTC TCG GGG ATC AG |  |  |  |  |  |  |
| Proliferative cells, including stem cells | SOX9 | F: CCT TGA GAA GAC CGT AGA CAA AG | 500 | 60 | 77 | 90.9 | 0.993 | In house |
|  |  | R: ATT CGG TAC GAG TTG CCT TTA G |  |  |  |  |  |  |
| Paneth cells | LYZ | F: CAC CAT GAA GGT TCT CCT GAC | 500 | 58 | 82 | 93 | 0.989 | In house |
|  |  | R: CCA TCT GGC CAA ACA GAC C |  |  |  |  |  |  |
| Enteroendochrine cells | CGA | F: CTG CGA GGA GAT GAA CGG AT | 400 | 60 | 83 | 101 | 0.990 | [6] |
|  |  | R: AGA ACC TCT GCG AGT TCG TC |  |  |  |  |  |  |
| Goblet cells | MUC2 | F: GAG TGT GAG TGG CAC TAC GAG | 400 | 59 | 83 | 95.3 | 0.989 | [6] |
|  |  | R: TCA TAG ATG GGC CTG TGC TTA |  |  |  |  |  |  |
| Tuft cells | DCLK1 | F: TGC TAC AGC ATC TAG TGT ACA GAA T | 500 | 58 | 81 | * |  | In house |
|  |  | R: CCT GCA TTC TTC GGA GCT G |  |  |  |  |  |  |
| IFN-β | IFN-β | F: CTCCACCACGGCTCTTTCTG | 400 | 60 | 81.5 | 94.5 | 0.994 | [43] |
|  |  | R: CTGCTGTGCTTGCTCAATCTC |  |  |  |  |  |  |
| IL-33 | IL-33 | F: ACA AGG AGC ACT CTG TGG A | 500 | 56 | 77 | 97.3 | 0.992 | In house |
|  |  | R: ACT CCT ATA AAC ACT CCA GGA T |  |  |  |  |  |  |
| TSLP | TSLP | F: TAG GGC TGG TGC TGA CCT AC | 400 | 55 | 77 | 96.7 | 0.995 | [44] |
|  |  | R: CCA CAG TAG ACA GTG TGG TTG AA |  |  |  |  |  |  |
| TLR2 | TLR2 | F: TGC TGC CAT TCT CAT TCT TC | 400 | 56 | 79 | 101.2 | 0.994 | [45] |
|  |  | R: GGG CCA CTC CAG GTA GGT |  |  |  |  |  |  |
| TLR5 | TLR5 | F: GGG CCT CTA CTC TGT TTC CA | 500 | 58 | 76.5 | 94.9 | 0.997 | [46] |
|  |  | R: CAC CAC CCG TGT CTA AGG AA |  |  |  |  |  |  |

*No serial dilution could be performed due to low expression.

1. Detournay O, Morrison DA, Wagner B, Zarnegar B, Wattrang E (2013) Genomic analysis and mRNA expression of equine type I interferon genes. J Interferon Cytokine Res 33(12):746-759.
2. Cvitas I, Galichet A, Chu Ling S, Müller EJ, Marti E (2020) Toll-like receptor-ligand induced thymic stromal lymphopoietin expression in primary equine keratinocytes. Vet Dermatol 31(2):154-e30.
3. Vendrig JC, Coffeng LE, Fink-Gremmels J (2013) Effects of separate and concominant TLR-2 and TLR-4 activation in peripheral blood mononuclear cells of newborn and adult horses. PLOS ONE 8(6):66897.
4. Kwon S, Gewirtz AT, Hurley DJ, Robertson TP, Moore JN, Vandenplas ML (2011) Disparities in TLR5 expression and responsiveness to flagellin in equine neutrophils and mononuclear phagocytes. J Immunol 186:6263-6270.
