# Supplementary figures and images for: Generation of equine enteroids and enteroid-derived 2D monolayers that are responsive to microbial mimics
Source: Vet Res. 2021 Aug 14;52:108. doi: 10.1186/s13567-021-00976-0 (PMC8364015; doi:10.1186/s13567-021-00976-0)

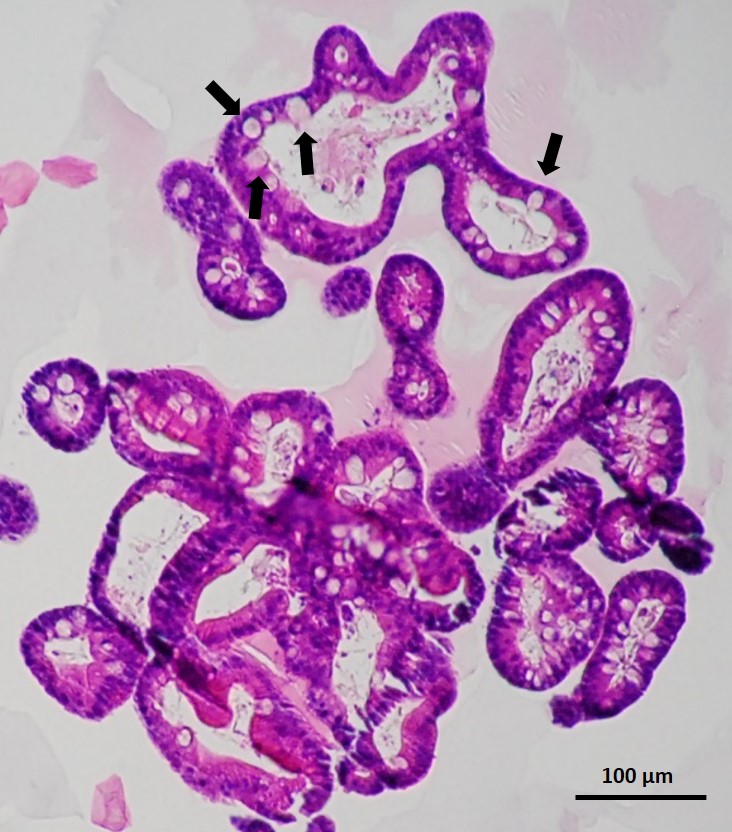

Supplement: Supplementary file 2 — Additional file 2. HE image of equine enteroids. HE stained section of equine enteroids at day 7 of culture. Arrows indicate goblet cells. [file 13567_2021_976_MOESM2_ESM.jpg]

**A**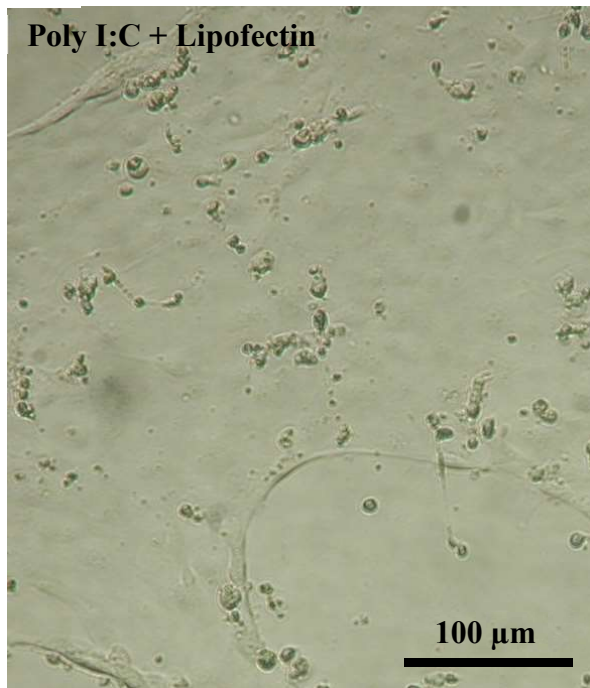**B**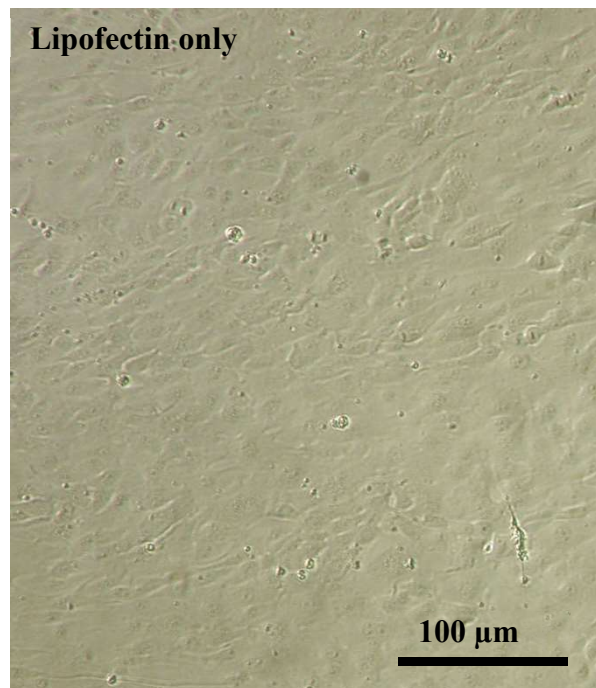**C**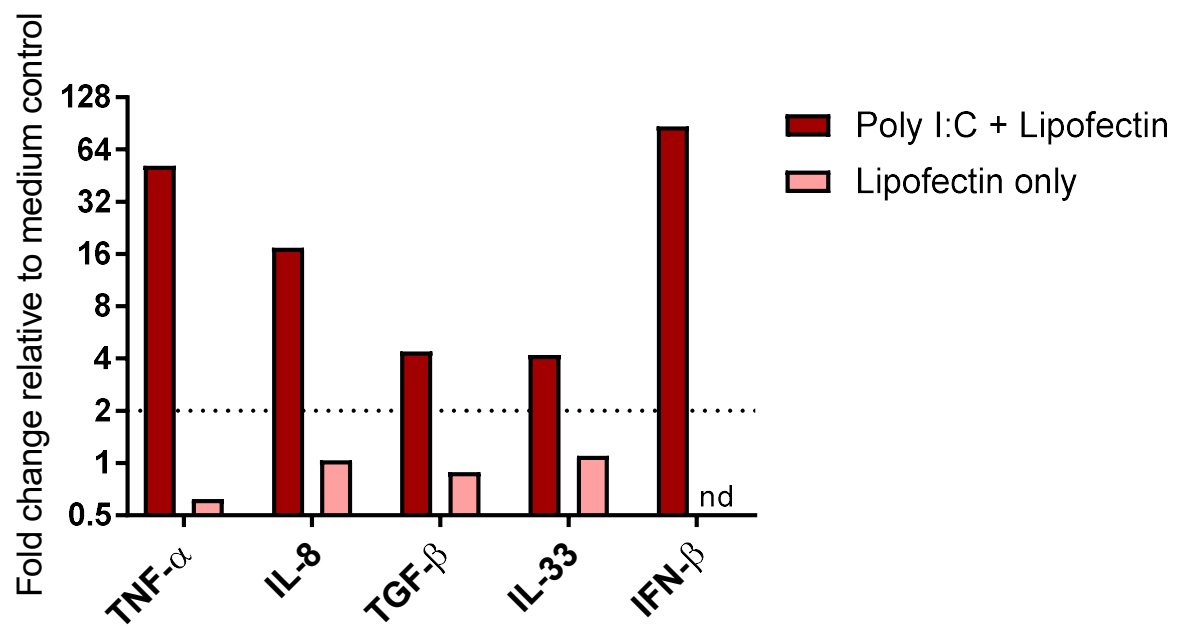

Supplement: Supplementary file 3 — Additional file 3. Equine enteroid-derived 2D monolayers exposed to Poly I:C + Lipofectin (A) or Lipofectin alone (B) and the relative expression of cytokine genes (C) after 20 h stimulation. The figure shows data from one representative horse (Photo: Nikon Coolpix 990). [file 13567_2021_976_MOESM3_ESM.pdf]
